# The Emerging Roles of the β-Secretase BACE1 and the Long Non-coding RNA BACE1-AS in Human Diseases: A Focus on Neurodegenerative Diseases and Cancer

**DOI:** 10.3389/fnagi.2022.853180

**Published:** 2022-03-21

**Authors:** Arezou Sayad, Sajad Najafi, Bashdar Mahmud Hussen, Sara Tharwat Abdullah, Ahmad Movahedpour, Mohammad Taheri, Mohammadreza Hajiesmaeili

**Affiliations:** ^1^Department of Medical Genetics, School of Medicine, Shahid Beheshti University of Medical Sciences, Tehran, Iran; ^2^Department of Medical Biotechnology, School of Advanced Technologies in Medicine, Shahid Beheshti University of Medical Sciences, Tehran, Iran; ^3^Department of Pharmacognosy, College of Pharmacy, Hawler Medical University, Erbil, Iraq; ^4^Center of Research and Strategic Studies, Lebanese French University, Erbil, Iraq; ^5^Department of Pharmacology and Toxicology, College of Pharmacy, Hawler Medical University, Erbil, Iraq; ^6^Behbahan Faculty of Medical Sciences, Behbahan, Iran; ^7^Institute of Human Genetics, Jena University Hospital, Jena, Germany; ^8^Skull Base Research Center, Loghman Hakim Hospital, Shahid Beheshti University of Medical Sciences, Tehran, Iran; ^9^Critical Care Quality Improvement Research Center, Loghman Hakim Hospital, Shahid Beheshti University of Medical Sciences, Tehran, Iran

**Keywords:** BACE1, BACE1-AS, lncRNA, Alzheimer’s disease, cancer

## Abstract

The β-Secretase (BACE1) is widely studied to be particularly involved in amyloid deposition, a process known as the pathogenic pathway in neurodegenerative diseases. Therefore, BACE1 expression is frequently reported to be upregulated in brain samples of the patients with Alzheimer’s disease (AD). BACE1 expression is regulated by BACE1-AS, a long non-coding RNA (lncRNA), which is transcribed in the opposite direction to its locus. BACE1-AS positively regulates the *BACE1* expression, and their expression levels are regulated in physiological processes, such as brain and vascular homeostasis, although their roles in the regulation of amyloidogenic process have been studied further. BACE1-AS dysregulation is reported consistent with BACE1 in a number of human diseases, such as AD, Parkinson’s disease (PD), heart failure (HF), and mild cognitive impairment. BACE1 or less BACE1-AS inhibition has shown therapeutic potentials particularly in decreasing manifestations of amyloid-linked neurodegenerative diseases. Here, we have reviewed the role of lncRNA BACE1 and BACE1-AS in a number of human diseases focusing on neurodegenerative disorders, particularly, AD.

## Introduction

Although non-coding RNAs (ncRNAs) have been paid less attention compared to protein-coding transcripts, studies in the past decades have unveiled their regulatory roles and widespread biological functions (Hussen et al., 2021b; Najafi et al., 2022b). Long ncRNAs (lncRNAs) are a group of ncRNAs, which are characterized by their length composed of more than 200 nucleotides, and so longer than other ncRNAs (Najafi et al., 2022a). Structurally, lncRNAs can be subdivided into linear and circular transcripts, of which the former class is mainly referred to as conventional transcripts, although each linear transcript can have a circular counterpart, and the latter structures are occasionally found with higher concentration and stability compared to their linear counterparts (Rahmati et al., 2021). They do not possess open reading frames (ORFs) [though exceptions have been reported (Bánfai et al., 2012; Hartford and Lal, 2020)] and thus predominantly not transcribed to protein-encoding transcripts; although studies have revealed protein-encoding potentials for a number of lncRNAs (Rinn and Chang, 2012), however, thousands of genes are identified in association with lncRNAs in mammalian genome, such as humans, which are transcribed to lncRNAs at high levels exceeding tens of thousands of lncRNAs in human cells (Guttman et al., 2009; Iyer et al., 2015). The expression of lncRNAs is mainly conducted from unconventional sequences, such as regulatory promoters, enhancers, sense and antisense transcripts, and introns from eukaryotic genomes (Al-Tobasei et al., 2016). Accordingly, lncRNAs have been divided into 8 subclasses based on their expressing genomic regions, including intergenic, intronic, promoter, enhancer, sense/antisense, bidirectional, small nuclear RNA, and non-polyadenylated lncRNAs (Ma et al., 2013). They are extensively found in all types of eukaryotic cells with specific patterns of expression making some transcripts limited to a specific type of cell or tissue or even a stage of organ development (Sarropoulos et al., 2019). Particularly, sequencing of some lncRNAs has shown that their sequences, although less compared to mRNAs, are conserved among different species, suggesting substantial roles for these non-coding transcripts, which has forced them to evolutionary pressure (Hezroni et al., 2015; Quinn and Chang, 2016). Although RNA sequencing studies have shown that a majority of lncRNAs demonstrate poor DNA conservation with specific expression in primates (Fang and Fullwood, 2016), RNA polymerase II (RNA Pol II) plays the main role in the transcription of lncRNAs, and phosphorylation of its carboxy-terminal domain can affect the lncRNAs expression (Statello et al., 2021). Although this mechanism controls the expression of lncRNAs, however, some lncRNAs escape this regulatory mechanism to be transcribed (Schlackow et al., 2017). Mechanisms responsible for the biogenesis of lncRNAs are not fully elucidated; however, several mechanisms, such as RNase cleavage, RNA and protein complexes recruiting to the ends of transcripts, generation of circular forms, and paraspeckles, are known to be involved in this process (Dahariya et al., 2019). LncRNAs are predominantly located at the cell nucleus and undergo several processes, such as splicing, although with lower efficiency compared to mRNAs, 5′ capping, and 3′ polyadenylation similar to already known protein-encoding mRNAs transcripts (Statello et al., 2021). Although enough evidence is still required to confirm the functionality of a majority of lncRNAs (Statello et al., 2021), however, current findings on a number of lncRNAs support that acting in both *cis* and *trans* pathways, lncRNAs play a role in the landscape of cell biology *via* regulation of a wide variety of substantial biological processes, such as cell proliferation and differentiation, organogenesis, gene expression, and epigenetic regulation (Kazemzadeh et al., 2015). Some of the best-known lncRNAs include X-inactive-specific transcript (XIST), which is known to contribute to epigenetic dosage compensation *via* regulation of X-chromosome inactivation (Cerase et al., 2015), Kcnq1ot1 in genomic imprinting, and a handful of lncRNAs, such as H19, MALAT1, and HOTAIR, with regulatory functions on cell cycle, proliferation, and tumorigenesis. Deregulation of these processes is already identified in association with pathological conditions, and thus, it is not surprising to know that an increasing number of lncRNAs show deviations in their regulation in association with various human diseases. These include neurodegenerative disorders, diabetes, hereditary diseases, coronary diseases, autoimmune disease, and various types of cancers (Wapinski and Chang, 2011; Sparber et al., 2019; Hussen et al., 2021a). The increasing importance of lncRNAs’ role in the landscape of human diseases has caused the requirement to databases, such as LncRNADisease, for collecting and providing access to findings on the association of lncRNAs with various diseases (Bao et al., 2019). Elucidation of the role of lncRNAs in the development of health disorders can help develop diagnostic and therapeutic approaches for various human disorders. In this study, although we outlined the roles of both BACE1 β-Secretase and its antisense transcript BACE1-AS in a number of human diseases, we have tried to focus on the regulatory role of BACE1-AS in the development of human diseases and its potentials as a biomarker.

In the amyloid cascade, the β-Secretase-1 (BACE1) enzyme is responsible for the cleavage of amyloid precursor protein (APP) resulting in the formation of amyloid-β 1 - 40 (Aβ1 - 40) and Aβ1 - 42, followed by secretase cleavage (Goedert and Spillantini, 2006). This process plays a central role in the pathophysiology of Alzheimer’s disease (AD), in which the biogenesis of amyloid plaques is responsible for the disease manifestations. Changes, either increased or decreased, in BACE1 expression have been associated with pathological conditions, such as AD, memory loss, and disturbance in synaptic plasticity, indicating strict regulatory mechanism controlling its expression (Faghihi et al., 2008). The regulation of BACE1 by a lncRNA was reported for the first time by Faghihi et al. (2008). This lncRNA is a natural antisense transcript transcribed in the opposite direction to the BACE1 locus and thus called BACE1-AS. This strand was initially identified among thousands of sense/antisense transcripts in human cells showing conservation with mice in FANTOM assays (Engström et al., 2006). BACE1-AS has a 2 kb length transcribed from the opposite strand of the sense locus on chromosome 11 (11q 23.3) (Kandalepas and Vassar, 2014). Although the expression pattern of BACE1-AS in normal cells is not determined, however, a few studies have reported that BACE1-AS is expressed in human cells regulating the BACE1 expression unlike BACE1 which is known to be expressed in various cell types. These cells expressing BACE1-AS include neuronal cells with a considerable high number of studies devoted to assess the BACE1-AS role in the AD pathophysiology and myocardial cells (Greco et al., 2017; Li et al., 2019). BACE1-AS expression is shown to be involved in physiological processes, such as apoptosis and neurogenesis (Modarresi et al., 2011; Sadeghi et al., 2018). The regulatory effect of BACE1-AS on BACE1 expression is associated with the pathophysiology of a handful of human diseases, particularly, AD and cancers. Here, we have a glance at an increasing number of evidence reporting the role of lncRNA BACE1-AS in various diseases with a focus on neurodegenerative diseases and malignancies.

## Neurodegenerative Diseases

### Alzheimer’s Disease

The AD is the most common dementia disorder affecting 29 million people in the past decades of life (Cole and Vassar, 2008). The formation of senile plaques and neurofibrillary tangles are the characteristic findings described in brain autopsies (Flaten et al., 2014). The amyloid hypothesis as the most popular model described for AD pathogenesis introduced in the period of 1991–1992 (Beyreuther and Masters, 1991; Hardy and Allsop, 1991; Selkoe, 1991; Hardy and Higgins, 1992) states that Aβ deposition is responsible for AD pathogenesis and the corresponding disease manifestations through triggering a cascade damaging the neurons and synapses (Morris et al., 2014). Aβ is produced from APP, which normally acts as a transmembrane protein, in a process of sequential cleavages by secretase enzymes (Zhang et al., 2011). Although APP can be affected by α-secretase, in the amyloidogenic process, two other secretases play the main role. First, β-Secretase cleaves the APP producing β-carboxyl-terminal fragments (bCTFs) sequentially affected by γ-secretase and finally generating Aβ (Zhang et al., 2011; Zhao et al., 2020).

**FIGURE 1:**
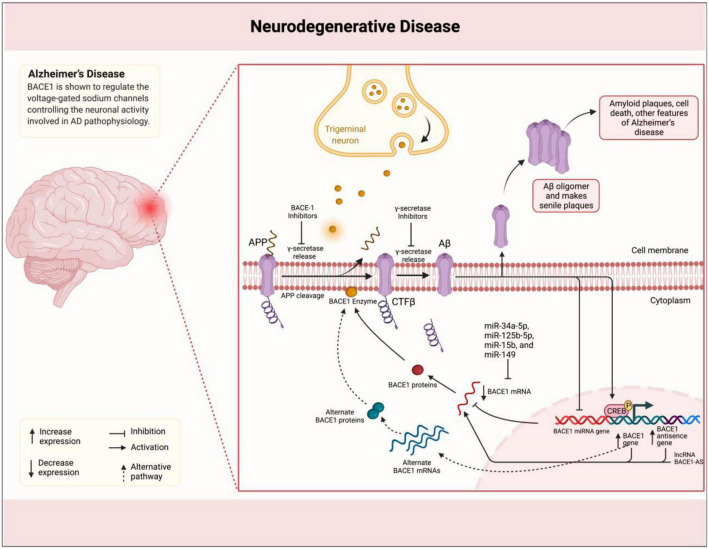
BACE1-AS promotes the synthesis of β-secretase in the brain of patients with AD. Amyloid plaques in the brain of patients with AD are linked to the illness. Plaques are formed by the successive breakdown of the APP *via* β-secretase (BACE1) and γ-secretase. In individuals with BACE1 deficiency, the mRNA is stabilized by binding to its antisense non-coding RNA (BACE1-AS). miRNAs, such as miR-34a-5p, miR-125b-5p, miR-15b, and miR-149, inhibit *BACE1* expression, decrease amyloid accumulation, and ameliorate neuronal injury.

Through APP cleavage and, consequently, generation of Aβ plaques, BACE1 plays a promoting role in the pathogenesis of AD (Cole and Vassar, 2008). BACE1 naturally plays a role in the processing of neuregulin-1 (NRG1), which is involved in development, and signaling pathways of neural cells (Fleck et al., 2012). Its levels have been reported to be elevated in brain samples of subjects with AD supporting other evidence indicating the association of BACE1 with AD pathogenesis (Sathya et al., 2012). BACE1 is shown to regulate the voltage-gated sodium channels controlling the neuronal activity involved in AD pathophysiology (Kim et al., 2007). Kandalepas et al. (2013) demonstrated that BACE1 expression is localized to the presynaptic terminals surrounding amyloid plaques in AD mouse models. Accordingly, Luo et al. (2001) showed that BACE1-deficient mice exhibit healthy phenotype and suppressed production of Aβ. Following advances in understanding the BACE1 mechanisms of action, several inhibitors have been suggested with potential application in AD treatment *via* targeting the enzymatic activity in AD development (Vassar and Kandalepas, 2011; Sathya et al., 2012). The lncRNA BACE1-AS has been shown to be upregulated in AD specimens by positively regulating the BACE1 expression using a feed-forward mechanism (Faghihi et al., 2008; Figure 1). Liu et al. (2014) showed that exogenous Aβ1–42 induces the expression of both BACE1 and BACE1-AS in the SH–SY5Y cell model for AD. BACE1-AS was demonstrated to enhance the BACE1 mRNA stability *via* forming RNA duplexes. Conversely, siRNA-mediated BACE1-AS knockdown caused to diminish in BACE1 potential for APP cleavage and formation of senile plaques. Furthermore, Zeng et al. (2019) in another study showed that BACE1-AS acts as a competing endogenous RNA (ceRNA) stabilizing the BACE1 expression *via* repressing several microRNAs (miRNAs) and targeting BACE1 mRNA. Accordingly, the levels of these miRNAs were increased upon BACE1-AS knockdown. Functionally, BACE1-AS is shown to promote autophagy contributing to neuronal damage (Zhou et al., 2021). Zhou et al. (2021) found that autophagy was increased consistent with BACE1-AS expression that led to neuron injury, while BACE1-AS knockdown improved damage injury *via* modulation of autophagy. The dual-luciferase assay revealed that BACE1-AS targets miR-214-3p and upregulates *ATG5* as a ceRNA. The same effect on BACE1-AS was observed when miR-214-3p was inhibited. These findings indicated that lncRNA BACE1-AS exerts its role in the development of AD phenotype through an axis mediated by miRNAs. This class of ncRNAs has been studied to act as players and suggested as biomarkers for several neurodegenerative and psychiatric disorders, such as AD, Parkinson’s disease (PD), and schizophrenia (Angelucci et al., 2019; Fyfe, 2020; Davarinejad et al., 2021), and, therefore, it is not surprising to see miRNAs involved in BACE1-AS-mediated pathogenesis in AD. Accordingly, another miRNA miR-214-3p was shown to perform the BACE1-AS impact on enhancing the isoflurane-induced neurotoxicity in AD SK-N-SH and SK-N-AS cell models (He et al., 2020). Taken together, an increasing number of evidence clearly confirms the role of lncRNA BACE1-AS in the pathophysiology of AD *via* regulation of BACE1 expression. This function has suggested BACE1-AS with diagnostic and therapeutic applications. Repeatedly, BACE1-AS has been reported to serve as a biomarker in the differentiation of patients with AD from healthy individuals. Through the comparison of BACE1-AS mRNA levels in plasma and plasma-derived exosomes retrieved from patients with AD and healthy controls, Fotuhi et al. (2019) found that although no association can be observed between BACE1-AS expression and state of disease, BACE1-AS showed higher expression in patients with AD compared to those at the pre-AD stage. In addition, receiver operating characteristic (ROC) curve analysis showed good sensitivity and specificity of BACE1-AS in the differentiation of patients with pre-AD and AD from healthy individuals. BACE1-AS in combination with traditionally used paraclinical diagnostic parameters has also shown an improving effect on diagnostic power (Wang et al., 2020). In this study, Wang et al. (2020) demonstrated that BACE1-AS levels in plasma exosomes in combination with right entorhinal cortex volume and thickness promote sensitivity and specificity of both parameters to 90.91 and 96.15%, respectively, in the detection of patients with AD compared to the condition when MRI parameters are used alone. Furthermore, targeting BACE1-AS has shown therapeutic potentials in improving AD symptoms and pathological manifestations in cell and animal studies. For instance, Modarresi et al. (2011) demonstrated that BACE1-AS knockdown ameliorates Aβ-associated hippocampal neurogenesis. Improved memory and learning behaviors in siRNA-mediated BACE1-AS-silenced AD mouse models were reported in another study described by Zhang et al. (2018). Ge et al. (2020) also found that BACE1-AS depletion in combination with berberine ameliorates Aβ_25–35_-associated neuronal injury in human primary neuron and SK-N-SH AD model cells. Curcumin as a polyphenol extracted from plant *Curcuma longa* Linn. has also shown a repressing effect on *BACE1* expression in SH-SY5Y cell lines, suggesting therapeutic potentials for AD (Huang et al., 2020). The same effect on amyloidogenesis has been reported for ginsenoside or combined taxifolin and cilostazol (Cao et al., 2016; Park et al., 2016). Bioinformatics analyses have also demonstrated therapeutic potentials for Lupeol and several compounds extracted from *Cajanus cajan* and *Citrus reticulata* by the inhibition of BACE1 *in situ* (Koirala et al., 2017; Adewole and Ishola, 2021). Moreover, miRNAs, such as miR-34a-5p, miR-125b-5p, miR-15b, and miR-149, have been demonstrated in distinctive studies to inhibit *BACE1* expression, decrease amyloid accumulation, and ameliorate neuronal injury (Gong et al., 2017; Li P. et al., 2020; Du et al., 2021). Overall, although the precise mechanism of action is not elucidated, however, BACE1-AS as a lncRNA is reportedly involved in the pathophysiology of AD rather than another disease, which is believed to play a role *via* the regulation of mRNA stability and expression of BACE1 β-Secretase. Accordingly, diagnostic and therapeutic potentials of BACE1-AS in AD have been paid more attention compared to other diseases hopefully suggesting that this lncRNA can bring beneficial potentials, particularly, in diagnosis and treatment of this disease.

### Parkinson’s Disease

The PD is the second common neurodegenerative disease after AD. PD is characterized by motor manifestations, such as tremor, rigidity, and bradykinesia (Schneider et al., 2017). Several lncRNAs have been found to be dysregulated in the PD pathophysiology, such as MALAT1 through facilitating the inflammasome activation (Cai et al., 2020), BDNF-AS and NEAT1 *via* promoting autophagy and apoptosis *in vitro* and *in vivo* (Fan et al., 2020; Dong et al., 2021), and SNHG1 *via* promoting neuronal injury (Wang H. et al., 2021). BACE1 and its antisense transcript BACE1-AS have been less studied in PD compared to those in AD. Bekris et al. (2015) reported a correlation between two single nucleotide polymorphisms in the *APP* gene and Aβ42 levels in cerebrospinal fluid (CSF) of patients with PD. Lange et al. (2015) also demonstrated an association between rs638405 polymorphism in the *BACE1* gene and enhanced risk of PD in a Norwegian population of patients with PD. The role of lncRNA BACE1-AS in PD was reported by Li Y. et al. (2020) in 2019, although, currently, further investigations are required to suggest BACE1-AS potentials in PD. In the latter study, the authors showed that BACE1-AS silencing ameliorates dopamine-dependent oxidative stress in PD model rats. The isolated substantia nigra tissues in rats with downregulated BACE1-AS had decreased α-synuclein and inducible nitric oxide synthase (iNOS). Diminished BACE1-AS also repressed neuronal injury and apoptosis in animals. The promoting effect of BACE1-AS on *BACE1* expression was shown to be exerted through “sponging” miRNA miR-34b-5p.

### Cancer

Today, cancer is among the most common causes of human diseases and related deaths with 19 newly diagnosed patients and 10 million deaths reported in 2020 (Ferlay et al., 2018). Several features are required to be achieved for the development of cancer known as “hallmarks of cancer.” These include limitless proliferative potential, self-sufficiency in growth signals, insensitivity to anti-growth signals, tissue invasion and metastasis, evading apoptosis, and sustained angiogenesis (Su et al., 2019). Several classes of ncRNAs, including lncRNAs, miRNAs, and circular RNAs, have been widely studied in various human malignancies in association with cancer development and progression (Visone and Croce, 2009; Huarte, 2015; Sayad et al., 2022). Although the precise functions of ncRNAs leading to cancer promotion are not completely elucidated, circRNAs, for instance, are found to regulate critical cellular processes *via* “sponging” miRNAs within axes of action leading to enhanced malignant behaviors (Taheri et al., 2021).

**FIGURE 2:**
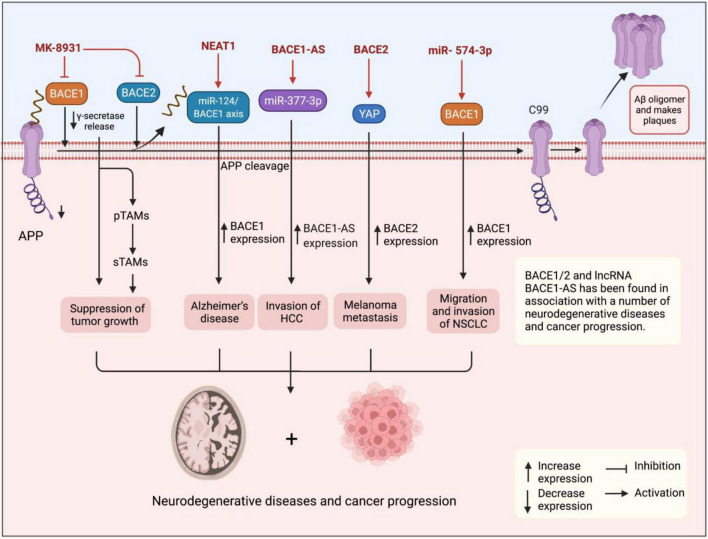
Figure shows the upregulation of BACE1 and BACE1-AS in cancer tissues and cells suggesting them with oncogenic functions. BACE1 and lncRNA BACE1-AS have also been found in association with progression for several cancers and neurodegenerative diseases. Pharmacological inhibition of BACE1 using MK-8931 causes reprogramming of pTAMs into sTAMs and suppression of tumor growth in glioblastoma.

Although BACE1 and BACE1-AS have been extensively studied in patients with AD, however, their role in tumorigenesis is poorly understood. The putative role of β-secretase BACE1 and its antisense transcript BACE1-AS in angiogenesis has suggested that they can play a role in tumorigenesis (Esfandi et al., 2019). It is also found to regulate signaling pathways involved in tumorigenesis (He et al., 2014). The APP plays a role in regulating cell proliferation in cancer cells, particularly, in breast cancer (Lim et al., 2014). Accordingly, elevated β-secretases BACE1 and BACE2 have been shown to play a role in a number of human malignancies (Farris et al., 2021). They were reported for the first time to enhance cell survival in pancreatic cancer cells, and also the effects of several inhibitors were compared with suppression of cell survival (Peters et al., 2012). Through affecting tumor microenvironment features, such as neutrophil extracellular traps (NETs), BACE1/2 leads to promoted cancer development and progression and, therefore, it is not surprising to see that their inhibition causes and modulates tumor growth (Munir et al., 2021). In another study, Zhai et al. (2021) very recently have demonstrated that pharmacological inhibition of BACE1 using MK-8931 causes reprogramming of tumor-promoting macrophages (pTAMs) into tumor-suppressive macrophages (sTAMs) and suppression of tumor growth in glioblastoma and, therefore, using AD drugs in the treatment of human cancers can be predicted (Martinez-Usatorre and De Palma, 2021; Figure 2). Conversely, miR-574-3p is shown to promote the migratory and invasive potentials of non-small cell lung cancer (NSCLC) cells (Yuan et al., 2019), which also particularly suggests potentials in fighting against human malignancies where silencing miRNAs using antisense oligonucleotides have shown a potent effect in cancer therapy (Dean and Bennett, 2003). Esfandi et al. (2019) reported downregulation of both *BACE1* and *BACE1-AS* in 30 tissue samples of patients with gastric cancer in consistent with findings for *BACE1* in breast ductal carcinoma (Yaghoobi et al., 2019).

Similar to BACE1, the lncRNA BACE1-AS has also been found in association with a number of cancers in a series of evidence. For instance, Wang M. et al. (2021) using expression patterns and survival data in 32 cancer types retrieved from the cBioPortal^1^ and Cancer Genome Atlas^2^ found that BACE1-AS is significantly upregulated in various human malignancies, such as glioblastoma, liver hepatocellular carcinoma (HCC), kidney renal clear cell carcinoma, and colon adenocarcinoma. Moreover, high BACE1-AS expression was correlated with poor survival in head and neck squamous cell carcinoma and liver carcinoma. BACE1-AS also showed a mutation in 0.9% of cancers mainly in melanoma cases. Importantly, according to the databases, BACE1-AS demonstrated a negative correlation with the tumor microenvironment. Experimentally, Liu et al. (2021) have recently reported the role of BACE1-AS in promoting the malignant features of HCC cells. They found that BACE1-AS expression is elevated in YY-8103, MHCC-97L, MHCC-97H, HCCLM3, and Huh-7 HCC cell lines compared to L02 healthy hepatic cell line and also in cancer tissues isolated from patients diagnosed with HCC compared to healthy adjacent tissues in the quantitative real-time polymerase chain reaction (qRT-PCR) assay, which was consistent with *in situ* findings. The dual-luciferase assay demonstrated that BACE1-AS sponges miR-377-3p confirming the bioinformatics predictions. Importantly, BACE1-AS knockdown suppressed the migration and invasion of HCC cells in wound healing and transwell assays, while BACE1-AS overexpression reversed the inhibitory effect on migratory and invasive potentials of cancer cells. Moreover, experiments in xenograft animal models revealed that BACE1-AS knockdown also inhibits tumor invasion and metastasis *in vivo*. Accordingly, elevated expression of BACE1-AS in cancer cells and tissues in addition to its promoting effects on their malignant features suggests BACE1-AS as an oncogenic lncRNA. In another study, Chen et al. (2016) demonstrated that BACE1-AS is responsible for antiproliferative and anti-invasive effects of anisomycin on ovarian cancer cells, while its knockdown ameliorates the chemotherapy-associated tumor suppressor effects and so concluded that BACE1-AS is a target for anisomycin in suppression of cancer cell proliferation and invasion. More than the evaluation of BACE1-AS potentials in cancer development and progression, this lncRNA has been studied as a biomarker with potential applications in the differentiation of several human cancers. For example, Nie et al. (2020), by analyzing the RNA-Seq expression data retrieved from the Cancer Genome Atlas database for 370 patients, found that the expression levels of upregulated BACE1-AS in liver cancer tissues were associated with clinicopathological features, such as tumor histological types and age of patients. BACE1-AS was shown to differentiate cancerous patients with high sensitivity, specificity, and area under the curve (AUC) values of 0.94, 0.836, and 0.949, respectively. Moreover, high BACE1-AS expression predicted poor overall survival (OS) and recurrence-free survival (RFS) for patients with liver cancer. In another study, Esfandi et al. (2019) reported diagnostic power of 75% for BACE1-AS in 30 patients with gastric cancer, while Yeganeh et al. (2020) also reported that increased expression of BACE1-AS along with 4 other lncRNAs in 30 tissues of breast cancer suggested them as potential biomarkers for breast cancer. Taken together, studies show upregulation of BACE1 and BACE1-AS in cancer tissues and cells suggesting them with oncogenic functions. Furthermore, the lncRNA BACE1-AS has shown diagnostic potentials in the differentiation of cancer patients from healthy individuals and also between different types of cancer.

### Heart Failure

Heart failure (HF) is a major cardiac disease with more than 37 million people estimated with the disease with high morbidity and mortality in the patients (Ziaeian and Fonarow, 2016). In association with amyloidogenic disorders, cardiac amyloidogenesis occurs upon heart involvement in systemic amyloidogenesis (Banypersad et al., 2012). Cardiac accumulation of transthyretin deposits in the affected patients seen in amyloidosis due to transthyretin deposition (ATTR) cause clinical manifestations of HF (Kittleson et al., 2020). Other types of amyloidoses with cardiac involvement include hereditary amyloidosis resulting from mutations in several genes, i.e., transthyretin and fibrinogen, and senile systemic amyloidosis (Banypersad et al., 2012). The role and diagnostic potentials of lncRNAs in the landscape of HF have been evaluated in a number of studies (Pang et al., 2016; Xu et al., 2020; Zhao et al., 2021). Zhuang et al. (2021) showed that lncRNA OIP5-AS1 that is highly found in striated muscles endures regulation in heart development, and mice models showed more severe HF under induction by pressure overload compared to healthy controls. In another study, Zhao et al. (2021) demonstrated that lncRNA MALAT1 is significantly upregulated in serum samples of 57 patients with HF and 40 HF rat models with an AUC of 0.918 in the prediction of patients with HF. Interestingly, MALAT1 inhibition ameliorated the degree of myocardial injury, improved the lipid metabolism, decreased inflammation in the rat models, and also relieved myocardial injury in the H9C2 cells. Similar results have been reported for SOX2-OT in H9C2 cells where it’s silencing reduced cell injury *via* promotion of cell viability, suppression of apoptosis, and ameliorating productions of collagens (Tu et al., 2021). Unlike cardiac amyloidogenesis, amyloid deposits are not common in cardiac muscle tissue in patients with HF; however, the involvement of ncRNAs in HF may demonstrate their complex role in the disease pathogenesis. The effect of BACE1-AS on HF was shown in a study by Greco et al. (2017). The authors measured the expression of BACE1 and BACE1-AS in the left ventricle biopsies retrieved from 18 patients with ischemic HF. The qRT-PCR results showed the elevated expression of both BACE1 and BACE1-AS in patient tissues compared to matched controls similar to the findings for mice models. BACE1 overexpression caused accumulation of intracellular Aβ consistent with results in patients with HF. Interestingly, HAOEC cells or HL-1 cells overexpressing BACE1-AS demonstrated transcriptomic changes similar to those treated with Aβ, which were seen particularly on cell proliferation, apoptosis, inflammatory responses, and signaling pathways. Finally, BACE1-AS overexpression induced apoptosis in endothelial cells, which was reversed by its silencing. Overall, BACE1-AS can act as a potential target for treating HF since accumulated Aβ is observed in patients with HF and BACE1-AS reduces Aβ accumulation and BACE1 levels.

### Other Diseases

Rather than the role of BACE1 and the regulatory function of BACE1-AS on amyloidogenic process clearly involved in the pathogenesis of particularly neurodegenerative diseases discussed earlier, both BACE1 and BACE1-AS have been reported with dysregulation in a number of other diseases, which can’t be easily associated with the amylogenic processes. These can be interpreted as impacts of these couple on gene expression and proteins known to play a role in the pathogenesis of several human disorders. Ghafouri-Fard et al. (2020) reported increased expression of BACE1 in children with autism spectrum disorder (ASD) compared to healthy children. Furthermore, BACE1-AS expression was correlated with the patient’s age. BACE1 and BACE1-AS also demonstrated diagnostic power of 0.762, and 0.795, respectively, for AUC in the ROC curve. In another study, Zhong et al. (2007) evaluated the BACE1 levels in the CSF specimens of 45 patients with mild cognitive impairment (MCI) *via* ELISA and Western blot analysis. The results demonstrated that patients with MCI have elevated BACE1 expression levels and β-Secretase enzymatic activity compared to healthy controls, which are also significantly higher than patients with AD. Interestingly, these findings suggest that BACE1, which is traditionally considered as an AD biomarker, can be used in the differentiation of patients with MCI from AD individuals. In addition, Cai et al. (2012) reported the expression of BACE1 in mice retina, and to explore its role on retinal health, they developed BACE1^–/–^ knockout animals. The results demonstrated distinct thinning in the neural retina, increased apoptosis in nuclei, and increased lipofuscin in addition to thinning and atrophy at the retinal pigment epithelium (RPE) in favor of retinal pathology. Furthermore, BACE1 inhibition using β-secretase inhibitor IV (β-SI) was shown to promote angiogenesis in mice retinas. These findings revealed the role of BACE1 in eye health and its dysregulation can cause retinal pathogenesis *via* disturbance in vascular homeostasis and accumulation of aging lipofuscin pigments beyond its traditional roles in amyloidogenesis.

## Concluding Remarks

β-Secretase (BACE1) particularly plays a role in amyloid-linked neurodegenerative diseases and its expression is known to be upregulated in brain samples of patients with AD. BACE1 expression is regulated by BACE1-AS, a lncRNA, which is transcribed in the opposite direction to its locus. BACE1-AS positively regulates the *BACE1* expression and, therefore, their dysregulation is consistently reported in a number of human diseases, such as AD, PD, HF, and MCI. It is known that lncRNAs have been found with their regulatory functions on gene expression and their dysregulations are being increasingly reported with involvement in human diseases. Accordingly, enhanced expression of BACE1-AS is also reported particularly in amyloid-linked neurodegenerative diseases consistent with BACE1 expression. Both are reported with diagnostic potentials in the differentiation of some diseases, and although BACE1 inhibition has been further explored with therapeutical effects, however, BACE1-AS also suggests potentials. Taken together, detection of BACE1-AS or BACE1 as an easy, reliable, and minimally invasive approach with applications in diagnosis of particularly neurodegenerative diseases or their targeting as a therapeutic approach for diminishing amyloidogenesis requires further studies, guaranteeing their usages in the promotion of human health in future. Among the efficient approaches for targeting nucleic acids, antisense oligonucleotides may suggest potentials for inhibition of BACE1 or lncRNA BACE1-AS.

## Author Contributions

MT designed and supervised the study. SN wrote the draft and revised it. BH, SA, MH, AM, and AS collected the data and designed the figures and tables. All authors read and approved the submitted version.

## Conflict of Interest

The authors declare that the research was conducted in the absence of any commercial or financial relationships that could be construed as a potential conflict of interest.

## Publisher’s Note

All claims expressed in this article are solely those of the authors and do not necessarily represent those of their affiliated organizations, or those of the publisher, the editors and the reviewers. Any product that may be evaluated in this article, or claim that may be made by its manufacturer, is not guaranteed or endorsed by the publisher.

## Footnotes

1
http://www.cbioportal.org

2
https://portal.gdc.cancer.gov/
